# Characteristics and mid-term follow-up of COVID-19 patients with hematological diseases: a retrospective study from a French tertiary care hospital

**DOI:** 10.1038/s41408-021-00512-5

**Published:** 2021-07-14

**Authors:** Nicolas Vallet, Sylvie Chevret, Linda Feghoul, Lorea Aguinaga, Louise Bondeelle, Eleonore Kaphan, Rémi Bertinchamp, Juliette Soret, Camille Villesuzanne, Nathalie De Castro, Marie Sebert, David Boutboul, Etienne Lengline, Jean-Jacques Tudesq, Florence Rabian, Lionel Adès, Alienor Xhaard, Roberta Di Blasi, Emmanuel Raffoux, Lionel Galicier, Jérôme Le Goff, Constance Delaugerre, Anne Bergeron, Stéphanie Harel, Saint-Louis CORE group, Nathalie De Castro, Jérôme Le Goff, Constance Delaugerre

**Affiliations:** 1 Hematology Transplantation, APHP Hospital St Louis, Paris, France; 2 ECSTRRA Team, University of Paris, INSERM, UMR 1153 CRESS, Paris, France; 3 Biostatistics and Medical Data Department, Saint-Louis Hospital, AP-HP, Paris, France; 4 Virology Department, APHP, Saint-Louis Hospital, Paris, France; 5 Hemato-oncology Department, AP-HP, Saint-Louis Hospital, Paris, France; 6 Pneumology Department, APHP, Saint-Louis Hospital, Paris, France; 7 Department of Clinical Immunology, AP-HP, Saint-Louis Hospital, Paris, France; 8 Service de Médecine Interne, Hôpital Saint Joseph, Marseille, France; 9 Center of Clinical Investigation, APHP, Saint-Louis Hospital, Paris, France; 10 Immunohematology Unit, APHP, Saint-Louis Hospital, Paris, France; 11 Infectious Disease Department, APHP, Saint-Louis Hospital, Paris, France; 12 Hematology Senior, APHP, Saint-Louis Hospital, Paris, France; 13 Hematology Adult, APHP, Saint-Louis Hospital, Paris, France; 14 Intensive Care Unit, APHP, Saint-Louis Hospital, Paris, France; 15 Hematology Adolescent and Young Adult Unit, APHP, Saint-Louis Hospital, Paris, France

**Keywords:** Haematological cancer, Infectious diseases

**Dear Editor**,

Coronavirus disease 2019 (COVID-19) is associated with an adverse impact on mortality among patients with hematological malignancies (HM) [1, 2]. Concern rise regarding immune response and safety of specific treatments in this population. We aimed to describe the characteristics and outcomes of patients followed in our institution, emphasizing on serological follow-up and treatment management strategies.

COVID-19 patients were prospectively recorded and defined according to the World Health Organization [3]. From March 1 to May 3, 2020, 143 patients were included. Among those, 128 (93%) were diagnosed with positive nasopharyngeal PCR SARS-CoV-2 while other showed evocative clinical of computerized tomography-scan (CT-scan) signs. Main characteristics and trajectories are detailed in Table 1 and Fig. 1a.

**Table 1:** Comparison of hematological disease and patient characteristics according to the care of SARS-CoV-2 infection.

	All included	Nosocomial	Secondary hospitalized	Outpatient	
*n*=	143	24	97	22	*p* value^a^
Age at COVID-19 diagnosis					
Continuous	66 (57–74)	70 (66–75)	67 (57–76)	56 (49–65)	0.002
>65 years	75 (52)	18 (75)	51 (52)	6 (27)	0.03
Sex					
Male	88 (61)	15 (62)	62 (63)	11 (50)	0.23
Comorbidities					
n/patients	3 (2–4)	3 (2–4)	3 (2–4)	1 (1–2)	<0.001
High blood pressure	63 (44)	15 (63)	44 (45)	4 (18)	0.02
Cardiovascular disease	40 (28)	9 (38)	28 (29)	3 (14)	0.18
Anticoagulants or anti-aggregants treatment	31 (22)	6 (25)	20 (21)	5 (23)	0.78
Smoker	28 (20)	8 (33)	20 (21)	0 (0)	0.03
Diabetes	20 (14)	3 (13)	16 (16)	1 (5)	0.19
Chronic respiratory insufficiency	19 (13)	6 (26)	12 (12)	1 (5)	0.46
Obesity	17 (12)	1 (4)	16 (16)	0 (0)	0.03
Chronic renal failure	18 (13)	5 (21)	13 (13)	0 (0)	0.12
Human immunodeficiency virus positive	3 (2)	1 (4)	2 (2)	0 (0)	0.99
*Hematological disease diagnosis*					0.68
Chronic lymphoid malignancy	79 (55)	13 (54)	55 (57)	11 (50)	
Non-Hodgkin lymphoma (NHL)	34	6	25	3	
Multiple myeloma (MM)	29	5	20	4	
Chronic lymphoid leukemia	12	2	9	1	
Hodgkin lymphoma	4	0	1	3	
Chronic myeloid malignancy	28 (20)	4 (17)	19 (20)	5 (23)	
Myelodysplastic syndromes	10	2	8	0	
Chronic myeloid leukemia	4	0	3	1	
Chronic myelomonocytic leukemia	2	0	2	0	
Other myeloproliferative neoplasm	12	2	6	4	
Acute Leukemia (AL)	17 (12)	6 (25)	10 (10)	1 (4)	
Acute myeloid leukemia	14	6	7	1	
Acute lymphoid leukemia	3	0	3	0	
*Allogeneic-HSCT*	9 (6)	0 (0)	7 (7)	2 (9)	
Non-malignant hematological disease	10 (7)	1 (4)	6 (6)	3 (14)	
Common variable immune deficiency	3	0	3	0	
Immune cytopenia	2	0	1	1	
Paroxysmal nocturnal hemoglobinuria	2	0	2	0	
Thrombotic thrombocytopenic purpura	1	1	0	0	
Aplastic anemia	1	0	0	1	
Castleman	1	0	0	1	
*Hematological disease status*					0.74
Disease without treatment	33 (23)	0 (0)	26 (27)	7 (31)	
Indolent, untreated	15	0	12	3	
Stable, last treatment >6 months	18	0	14	4	
Controlled disease under treatment	77 (54)	14 (58)	51 (52)	12 (55)	
Complete response in treatment	43	7	27	9	
Partial response in treatment	34	7	24	3	
Progressive disease	33 (23)	10 (42)	20 (21**)**	3 (14)	
Frontline	6	0	5	1	
Progressive disease with curative project	19	9	8	2	
Progressive disease without curative project	8	1	7	0	
Ongoing treatment					0.87
Never treated or Untreated in the last six months	39	0	32	7	
Continuous treatment	53	10	36	7	
Intermediate chemotherapy (NHL, triplet MM)	42	7	27	8	
Intensive chemotherapy (Auto-HSCT, AL)	9	7	2	0	
Hematological parameters before COVID-19, median (IQR)					
Absolute neutrophil count, 10^9^/L	2560 (1480–4440)	4000 (1355–6775)	2550 (1528–4788)	2790 (2040–3900)	0.45
Lymphocyte count, 10^9^/L	981.5 (535–1749)	720 (430–1253)	987 (500–1815)	1190 (810–1861)	0.37
Gamma globulins, g/L	7.4 (4.4–11.0)	8.7 (4.7–10.5)	7.7 (3.9–11.9)	7.4 (4.8–8.8)	0.80

Continuous variables are described as median with interquartile range.

*HSCT* hematopoietic stem cell transplantation.

^a^Statistical comparisons only deal with secondary hospitalized patients and outpatients.

**Fig. 1:**
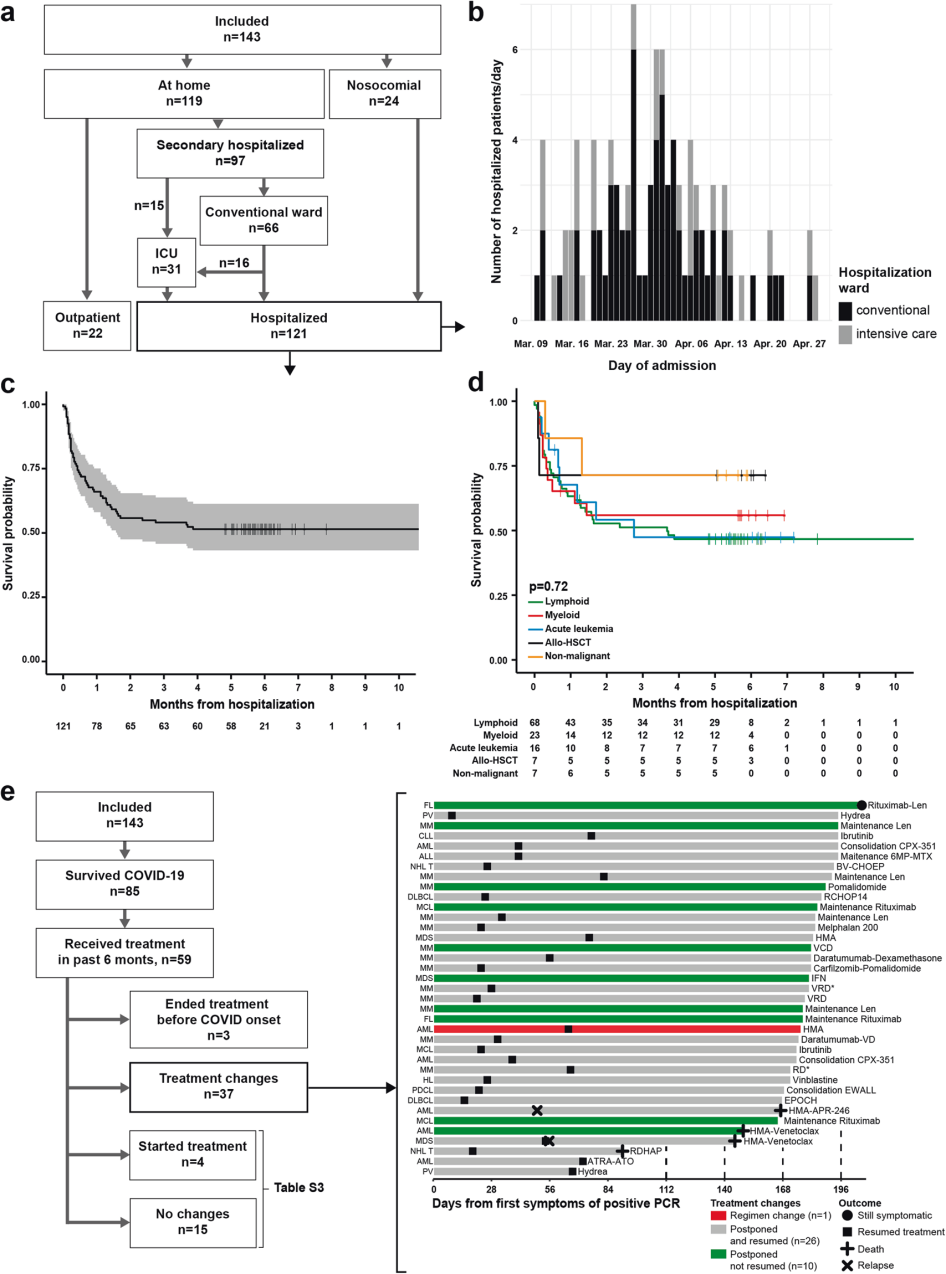
Trajectories and outcome of included patients. **a** Flowchart of trajectories of included patients. **b** Number of newly diagnosed patients per day during COVID-19 pandemic. **c** Kaplan–Meier curve for overall survival of the hospitalized patient cohort. **d** Kaplan–Meier curve comparing patients according to hematological disease categories. **e** Impact of COVID-19 on treatments schedules. Swimmer plot illustrating the treatment reintroduction and relapses among the COVID-19 survivors in which treatment schedules were changed. Each line represents one patient’s follow-up time, with the corresponding hematological disease and current treatment depicted at its left and right, respectively. AML acute myeloid leukemia, MDS myelodysplastic syndromes, MCL mantle cell lymphoma, NHL non-Hodgkin lymphoma, CLL chronic lymphoid leukemia, MM multiple myeloma, FL follicular lymphoma, HL Hodgkin lymphoma, PDCL plasmacytoid dendritic cell leukemia, DLBCL diffuse large B-cell lymphoma, PV polycythemia vera, HMA hypomethylating agents, Len lenalidomide, 6-MP mercaptopurine, MTX methotrexate, BV brentuximab-vedotin; *Only dexamethasone was postponed. ICU intensive care unit, allo-HSCT allogeneic hematopoietic stem cell transplantation (allo-HSCT).

Twenty-four (17%) patients were already hospitalized at the time of COVID-19, suggesting nosocomial contamination. Median time from admission to COVID-19 diagnosis was 22 days (IQR: 18-32, range 2–145). Fifteen patients (63%) were hospitalized in a conventional hematological ward and nine (37%) patients in re-education. Patients with HM have a tenfold higher incidence of nosocomial COVID-19 than patients without cancer [4].

In the 119 patients not previously hospitalized, hospitalization was required in 97 (68%) patients (Fig. 1a, b). This rate of hospitalization may not be representative of real-life data because COVID-19 in non-hospitalized patients was largely underestimated in March 2020 in France because of the absence of systematic COVID-19 testing. Secondary hospitalized patients were significantly older than outpatients, had higher number of comorbidities and higher frequency of hypertension, smoking habits and obesity [5]. There was no difference between hospitalized and non-hospitalized patients regarding remaining HD diagnosis, HD status or ongoing treatment (Table 1).

Thirty-one (26%) of the hospitalized patients required ICU supervision which is higher than described elsewhere, ranging from 6 to 11% [1, 6] (Fig. 1a, b). Among the 58 deceased patients, 19% were younger than 70 and were not transferred to the ICU. Most of them had progressive HM. Regarding the pandemic background, some decisions may have been influenced by the strain in bed availability. Still, the high rate of ICU admission compared with previous studies suggests that patients were prioritized, regardless of the HD [7]. No comorbidities besides body mass index (26.5 [IQR: 23–29] in those admitted to ICU vs. 24 [IQR: 21–27] in those who did not; *p* = 0.02) nor hematological disease were associated with ICU hospitalization risk.

Clinical, biological and radiological characteristics of the 121 hospitalized patients are reported in Table S1. Fever was not systematic but among the most frequent symptoms, along with respiratory signs [6, 8]. Digestive signs were found in 20% of patients, acute kidney injury (AKI) and liver injury in 22 and 3%, respectively. Seven patients had thrombosis, including four pulmonary embolism (Table S1). Three of these patients died. Of note, one patient with AL amyloidosis who received curative anticoagulation for pulmonary embolism died 3 weeks after of a subdural hematoma.

Blood SARS-CoV-2 RT-PCR was performed in 37 patients (Table S2). SARS-CoV-2 was detectable in 24 (65%) patients who had a higher number of comorbidities. The maximum median cycle threshold value was 34.7 (IQR: 33.4–36.3) and was not associated with HD categories (*p* = 0.40).

All patients received antibiotics. Systematic thrombo-embolic prophylaxis was first heterogeneous, then a higher dose was used from April 4, 2020, according to *Groupe Français d’études sur l’Hémostase et la Thrombose* (GFHT) recommandations [9]. Ten (11%) patients received different specific COVID-19 drug, thus no conclusion can be drawn from these data (Table S1).

We chose to restrict the outcome study to hospitalized patients. The median follow-up was 118 days (IQR: 13–175) since hospital admission. Survival rate was 54% (95% CI, 46–64%) (Fig. 1c). This is comparable with the previous study, reporting 60– 67% OS at 1 month [6]. A total of 58 (48%) patients died at 3 months. Of note, 53 (95%) patients died directly from COVID-19. Five patients died from their HM in the weeks following COVID-19. During the same period, the rate of mortality among patients without hematological diseases was 27% (*n* = 45/166, *p* < 0.001). This is consistent with previous studies showing an adverse impact of cancer on survival from COVID-19 [4].

In univariable analyses, factors hazard of death was associated with: age above 65 (HR = 2.76, 95% CI, 1.53–4.97), obesity (HR = 2.36, 95% CI, 1.24–4.47), cardiovascular disease (CVD) (HR = 2.56, 95% CI, 1.52–4.29), chronic kidney disease (HR = 2.06, 95% CI, 1.11–3.83), AKI (HR = 2.36, 95% CI, 1.36–4.09), oxygen supply >5 L/min (HR = 2.10, 95% CI, 1.21–3.64), and CRP levels >100 mg/L (HR = 2.02, 95% CI, 1.18–3.46). These results are consistent with non-cancer patients [5]. Multivariable Cox analysis selected five predictors: age above 65 (HR = 2.87, 95% CI, 1.52–5.42), obesity (HR = 2.58, 95% CI, 1.31–5.09), CVD (HR = 2.15, 95% CI, 1.26–3.68), oxygen supply >5 L/min (HR = 1.78, 95% CI, 1.00–3.17), and CRP >100 mg/L (HR = 1.96, 95% CI, 1.13–3.42). In the HM patients, there is no difference between lymphoid and myeloid malignancies. The impact of HD on mortality was heterogeneous in previous studies. Some report an impact of acute myeloid leukemia (AML) [1, 2], lymphoma [1] or myeloma [1] diagnosis on survival. In our cohort, there was no evidence of a prognostic value of HD status and type of ongoing treatment were not associated with OS. Ongoing treatment was also reported to be associated with mortality [10], notably with monoclonal antibodies [2] but a meta-analysis of 34 studies reported (i) similar risk of mortality among different HD and (ii) absence of impact of recent therapy in relative risk of death [11].

Immune responses were next assessed by serological assays retrospectively performed in 57 (41%) patients (Supplementary methods). Median time from COVID-19 diagnosis to first and last sample were 26 (IQR: 15–56) and 61 (IQR: 35–89) days. Seroconversion, defined by detection of blood SARS-CoV-2 IgG, was observed in 37 (65%) patients. The median time to seroconversion was 31 (IQR: 19–56) days. This rate is comparable to what has been observed in non-cancer patients, suggesting that pooled HD are not associated with a lower risk of seroconversion [12]. Seroconversion rate was not influence by HD categories (*p* = 0.40) but by anti-CD20 treatment in which the seroconversion rate was lowered to 14% (*n* = 1/7) compared to other 72% (*n* = 36/50, *p* = 0.005). This finding is consistent with reports of SARS-CoV-2 viremia persistent over 21 days in patients treated with rituximab [13]. Of note, one patient treated with rituximab and lenalidomide for a follicular lymphoma (FL) before COVID-19 developed a chronic COVID. Seven months after the infection, she still had clinical and CT-scan signs and a positive nasopharyngeal RT-PCR. All symptoms disappear after a treatment by COVID-19-convalescent plasma. Strategies using convalescent plasma may be helpful in this context [14]. This observation support the hypothesis that anti-CD20 therapies may lower the efficacy of the SARS-CoV-2 vaccine [15] thus these patients may not benefit of vaccines even with a third dose. Vaccination of their relatives should be a priority. When a COVID-19 infection is diagnosed, anti-COVID-19 monoclonal antibodies should be quickly discussed. Together, these results encourage physicians to carefully assess benefit/risk ratio notably in maintenance therapies.

SARS-CoV-2 IgG, titer follow-up was available in 16 patients (median time from diagnosis to last sample: 61 (IQR: 35-89) days). Only one patient with previous allo-HSCT treated with corticosteroid became undetectable for IgG at 83 days.

To evaluate the feasibility of reintroducing specific treatment in patients who survived from COVID-19, specific treatment modifications were analyzed in 59 patients who received at least one specific drug in the last 6 months before COVID-19 (Fig. 1e). Patients who started (*n* = 4) or continued a therapy (*n* = 15) had no adverse events or severe forms of COVID-19 (Table S3). Treatment schedules modifications were observed in the 37 (62%) patients. Regimen change was observed in one AML patient. For 36 patients, treatments were postponed. Among them, treatment resumed in 26 (72%) patients. The median time of delay from the theoretical date of next cycle was 32 (IQR: 23–63) days. None of the asymptomatic patients who resumed their treatments presented a signs of COVID-19, including two with persistent positive nasopharyngeal RT-PCR. At the last follow-up, treatment was not resumed in 10 (28%) patients with asymptomatic and controlled HD, according to the physician’s decision. Eight had stable diseases, one progressive disease and one patient was still COVID-19 symptomatic (described above).

To conclude, SARS-CoV-2 infections in patients with HD is not only associated with a dismal prognosis but also with treatment course changes that may impact disease’s evolution in the mid-term. Age, obesity, and CVD notably predicted death from COVID-19. Restart of previous treatment was not associated with a second hospitalization related to COVID-19. This may be explained by the 64% of patients who produced anti-SARS-CoV-2 IgG, suggesting that they might not be at higher risk of a second infection or worsening. Nevertheless, because lower seroconversion rate was observed in anti-CD20 treated patients, physicians should carefully assess benefit/risk ratio before introducing these drugs or when mandatory, carefully monitor these patients.

## Supplementary information

Supplementary Data
