# Supplementary material for: Characteristics and mid-term follow-up of COVID-19 patients with hematological diseases: a retrospective study from a French tertiary care hospital
Source: Blood Cancer J. 2021 Jul 14;11(7):129. doi: 10.1038/s41408-021-00512-5 (PMC8279108; doi:10.1038/s41408-021-00512-5)
Supplement: Supplementary file 1 — Supplementary Data [file 41408_2021_512_MOESM1_ESM.pdf]

Characteristics and mid-term follow-up of COVID-19 patients with hematological diseases: a retrospective study from a French tertiary care hospital

Nicolas Vallet, Sylvie Chevret, Linda Feghoul, Lorea Aguinaga, Louise Bondeelle, Eleonore Kaphan, Rémi Bertinchamp, Juliette Soret, Camille Villesuzanne, Nathalie De Castro, Marie Sebert, David Boutboul, Etienne Lengline, Jean-Jacques Tudesq, Florence Rabian, Lionel Adès, Alienor Xhaard, Roberta Di Blasi, Emmanuel Raffoux, Lionel Galicier, Jérôme Le Goff, Constance Delaugerre, Anne Bergeron, Stéphanie Harel, Saint-Louis CORE group.

SUPPLEMENTARY DATA

Supplementary methods

Table S1. Clinical, biological and radiological characteristics and outcome of hospitalized patients

Table S2. Comorbidities of patients included in the blood SARS-CoV-2 RT-PCR analysis

Table S3. Details of pursued treatments at time of COVID-19

Saint Louis CORE group full list of members

Supplementary Methods.

RT-PCR and serological analyses

Serological analyzes were performed on the Abbott Architect instrument using the Abbott SARS-CoV-2 IgG assay after FDA notification following the manufacturer's instructions. The assay is a chemiluminescent microparticle immunoassay for the qualitative detection in serum or plasma of IgG against the SARS-CoV-2 nucleoprotein.

RT-PCR for the detection of SARS-CoV2 RNA for the diagnosis and for the follow up of COVID-19 was performed using the Roche cobas® 6800 SARS-CoV-2 assay (Roche Diagnostics, Meylan, France) according to the manufacturer's instructions. The sample preparation is fully automated (nucleic acid extraction and purification) followed by PCR amplification and detection. The assay targets the ORF1 a/b nonstructural region that is unique to SARS-CoV-2 and the E-gene a conserved region in the structural protein envelope.

Table S1. Clinico-biological characteristics and outcome of hospitalized patients

	All included	Chronic lymphoid Malignancy	Chronic Myeloid Malignancy	Acute leukemia	Allo-HSCT	Non-malignant
n=	**121**	**68**	**23**	**16**	**7**	**7**
**Time from diagnosis to hospitalization (d)[a]**	5 (2-8)	5 (2-9)	7 (3-8)	3 (1-6)	5 (4-6)	7 (5-11)
**Clinical manifestations**						
Asymptomatic	4 (3)	3 (4)	0 (0)	0 (0)	0 (0)	1 (14)
Fever	99 (82)	53 (78)	19 (83)	14 (88)	7 (100)	6 (86)
Dyspnea	75 (62)	45 (66)	16 (70)	6 (38)	5 (71)	3 (43)
Cough	69 (57)	36 (53)	14 (61)	6 (38)	7 (100)	6 (86)
Digestive	26 (21)	11 (16)	6 (26)	6 (38)	1 (14)	2 (29)
Diarrhea	19 (16)	9 (13)	5 (22)	3 (19)	0 (0)	2 (29)
Nausea, vomiting, abdominal pain	12 (10)	4 (6)	2 (9)	4 (25)	1 (14)	1 (14)
Chest pain	5 (4)	2 (3)	1 (4)	1 (6)	0 (0)	1 (14)
Anosmia and dysgeusia	10 (8)	7 (11)	2 (9)	0 (0)	0 (0)	1 (14)
Myalgia	10 (8)	6 (9)	3 (13)	0 (0)	0 (0)	1 (14)
Neurological	19 (17)	12 (19)	6 (26)	1 (6)	0 (0)	0 (0)
Acute Kidney Injury	27 (24)	16 (26)	8 (35)	1 (6)	1 (14)	1 (14)
Acute Hepatitis	4 (5)	2 (3)	0 (0)	2 (13)	0 (0)	0 (0)
Thrombosis	7 (6)	3 (4)	1 (4)	2 (13)	0 (0)	2 (14)
Pulmonary embolism	4	2	1	0	0	1
Other venous sites	3	1	0	2	0	0
**Blood biological parameters**						
Absolute neutrophil count (10^9/L)	2.44 (1.48-4.51)	3.06 (1.97-4.95)	1.03 (0.37-3.60)	0.98 (0.32-3.33)	2.75 (2.08-3.29)	1.96 (1.44-2.92)
Lymphocytes (10^9/L)	0.67 (0.40-1.10)	0.77 (0.40-1.70)	0.58 (0.47-0.82)	0.34 (0.28-0.66)	0.34 (0.27-0.69)	0.59 (0.50-0.77)
Gammaglobulinemia (g/L)	7.5 (4.4-11.6)	6.2 (3.6-10.9)	10.1 (9.3-11.7)	9.3 (7.9-12.1)	5.4 (4.8-6.7)	10.9 (10-12.5)
C-reactive protein (mg/L)	76 (36-146)	72 (36-137)	82 (55-158)	91 (63-255)	24 (11-110)	107 (33-128)
Fibrinogen (g/L)	6.1 (4.7-7.5)	6.1 (5.4-7.4)	6.7 (4.6-7.4)	4.9 (4.1-6.3)	7.0 (6.6-7.4)	6.2 (4.5-7.7)
Ferritin (µg/L)	1063 (421-2692)	588 (403-1760)	719 (252-852)	4867 (3074-5564)	4937 (2622-5980)	1622 (1368-1877)
D-Dimer (ng/mL)	1005 (632-2095)	868 (645-1110)	2115 (1192-3038)	3280 (2562-3998)	690 (560-1515)	965 (767-1163)
LDH (U/L)	557 (359-857)	511 (296-719)	601 (435-860)	756 (522-1184)	1005 (1001-1009)	-
**Computed tomography scan findings**						
Ground glass opacities	48 (40)	27 (40)	11 (48)	5 (31)	2 (29)	27 (43)
Condensation	34 (28)	19 (28)	8 (35)	3 (19)	3 (43)	1 (14)
Pleural effusion	8 (7)	4 (6)	3 (13)	1 (6)	0 (0)	0 (0)
**Co-infections**						
Bacterial	11 (10)	5 (7)	3 (13)	2 (13)	1 (14)	0 (0)
Fungal	3 (2)	0 (0)	2 (9)	0 (0)	1 (14)	0 (0)
Viral	3 (2)	2 (3)	0 (0)	0 (0)	1 (14)	0 (0)
**Treatments**						
O_2						
Standard O_2	62 (51)	32 (47)	15 (65)	9 (56)	4 (57)	2 (29)
Mechanical ventilation	24 (20)	19 (28)	2 (9)	2 (13)	0 (0)	1 (14)
ICU admission	31 (26)	23 (34)	3 (13)	2 (13)	1 (14)	2 (29)
Frontline ICU admission	15 (12)	10 (15)	3 (13)	1 (6)	0 (0)	1 (14)
**COVID-19 specific treatment**						
Lopinavir or equivalent	11 (10)	4 (7)	5 (22)	1 (6)	0 (0)	1 (14)
Dexamethasone	7 (6)	4 (7)	0 (0)	1 (6)	0 (0)	2 (33)
Hydroxychloroquine	5 (4)	2 (3)	0 (0)	1 (6)	2 (29)	0 (0)
Tocilizumab	4 (3)	3 (5)	0 (0)	1 (6)	0 (0)	0 (0)
Eculizumab	4 (3)	2 (3)	0 (0)	0 (0)	1 (14)	1 (14)

Table S2. Comorbidities of patients included in the blood SARS-CoV-2 RT-PCR analysis

	Undetectable viremia	Detectable viremia	
n=	13	24	p-value
**Age at COVID-19 diagnosis**			
continous, median (IQR)	62 (54-69)	61 (57-70)	0.87
>65 years	5 (38)	10 (42)	0.99
>70 years	2 (15)	6 (25)	0.68
**Sex**			
Male	8 (62)	15 (63)	0.99
**Comorbidities**			
n/patients	3 (2-3)	3 (2-4)	0.62
High blood pressure	6 (46)	10 (42)	0.99
Cardiovascular disease	1 (8)	8 (33)	0.11
Anticoagulants or antiaggregants treatment	1 (8)	4 (17)	0.63
Smoker	3 (23)	4 (17)	0.68
Diabetes	0 (0)	4 (17)	0.28
Chronic respiratory insufficiency	2 (15)	6 (25)	0.69
Obesity	1 (8)	7 (29)	0.22
Chronic renal failure	1 (8)	0 (0)	0.35
Human immunodeficiency viruse positive	2 (15)	0 (0)	0.12
**Hematological disease diagnosis**			
**Chronic lymphoid malignancy**	**8 (62)**	**14 (58)**	0.75
Non-Hodgkin lymphoma (NHL)	7	8	
Multiple myeloma (MM)	0	6	
Chronic lymphoid leukemia	1	0	
**Chronic myeloid malignancy**	**1 (8)**	**2 (8)**	
Myelodysplastic syndromes	1	1	
Chronic myeloid leukemia	0	1	
**Acute Leukemia**	**1 (8)**	**5 (21)**	
Acute myeloid leukemia	0	1	
Acute lymphoid leukemia	1	4	
**Allogeneic-HSCT**	**2 (15)**	**1 (4)**	
**Non-malignant hematological disease**	**1 (8)**	**2 (8)**	
Common variable immune deficiency	0	1	
Paroxysmal nocturnal hemoglobinuria	0	1	
Thrombotic thrombocytopenic purpura	1	0	

HSCT: hematopoietic stem cell transplant.

Table S3: Details of pursued treatments at time of COVID-19

Treatment schedules	Hematological disease	Hospitalized	Administered treatment
Started treatment	Chronic lymphoid leukemia (hemolytic auto-immune anemia)	Yes	prednisone
	Diffuse large B cells lymphoma	Yes	R-CHOP (full dose)
	Chronic myeloid leukemia	Yes	hydroxyurea, nilotinib
	Refractory immune thrombocytopenia	Yes	dexamethasone, immunoglobulin, vinblastin, romiplostim
Unchanged treatment	Post-HSCT	Yes	sirolimus
	Post-HSCT	Yes	prednisone
	Post-HSCT	Yes	prednisone, ciclosporine, imatinib
	Chronic myeloid leukemia	No	nilotinib
	Myelodysplastic syndrome	Yes	CPX-351 57 days before COVID-19, waiting for HSCT
	Primitive myelofibrosis	Yes	ruxolitinib
	Multiple myeloma	Yes	revlimid (maintenance)
	Polycytemia vera	No	interferon
	Polycytemia vera	No	hydroxyurea
	Polycytemia vera	Yes	ruxolitinib
	Polycytemia vera	No	ruxolitinib
	Essential thrombocytemia	No	ruxolitinib
	Essential thrombocytemia	Yes	hydroxyurea
	Castleman disease	No	tocilizumab
	Paroxysmal nocturnal hemoglobinuria	Yes	eculizumab

HSCT: hematopoietic stem cell transplant.

Saint Louis CORE group full list of members

The Saint Louis CORE group is a collaborating group of clinicians, radiologists, biologists, pharmacists and clinical research assistants of Saint Louis Hospital. They all have participated to the care of patients with COVID19 and/or to research into COVID19 in Saint Louis Hospital, Paris, during the SARS-COV2 epidemic. They decided to share their data to ease local research into COVID19. All the manuscript written on behalf of the Saint Louis CORE group has been, preliminary to submission, sent to all members for critical rereading and consent for publication.

The members of the Saint Louis CORE group are:

Achilly Y,
Ades, L
Aguinaga L,
Archer G,
Benattia A,
Bercot B,
Bergeron A,
Bertinchamp R,
Bondeelle L,
Bouaziz JD,
Bouda D,
Boutboul D,
Brindel Berthon I,
Bugnet E,
Caillat Zucman S,
Cassonet S,
Celli Lebras K,
Chabert J,
Chaix ML,
Chevret S,
Clément M,
Davoine C,
De Castro N,
De Kerviler E,
De Margerie-Mellon C,
Delaugerre C,
Depret F,
Denis B,
Djaghout L,
Dupin C,
Farge-Bancel D,
Fauvaux C,
Feghoul L,
Fenaux H,
Feredj, E,
Feyeux D,
Fontaine JP,
Fremeaux-Bacchi V,
Galicier L,
Garestier J,
Harel S,
Jegu AL,
Kozakiewicz E,
Lebel M Baye A,
Le Goff J,
Le Guen P,
Lengline E,
Liegon G,
Lorillon G,
Madelaine Chambrin I,
Mahjoub N,
Martin de Frémont G,
Maylin S,
Mehlman C,
Meunier M,
Molina JM,
Oksenhendler E,
Peffault de la Tour R,
Peyrony O,
Plaud B,
Rouveau M,
Salmona M,
Saussereau J,
Schnepf N,
Soret J,
Tazi A,
Thegat M,
Tremorin MT.
